# Reprogrammable plasmonic topological insulators with ultrafast control

**DOI:** 10.1038/s41467-021-25835-6

**Published:** 2021-09-15

**Authors:** Jian Wei You, Qian Ma, Zhihao Lan, Qiang Xiao, Nicolae C. Panoiu, Tie Jun Cui

**Affiliations:** 1 Department of Electronic and Electrical Engineering, University College London, London, UK; 2 State Key Laboratory of Millimeter Waves and Institute of Electromagnetic Space, Southeast University, Nanjing, China

**Keywords:** Electrical and electronic engineering, Metamaterials, Photonic crystals, Photonic devices

## Abstract

Topological photonics has revolutionized our understanding of light propagation, providing a robust way to manipulate light. So far, most of studies in this field are focused on designing a static photonic structure. Developing a dynamic photonic topological platform to switch multiple topological functionalities at ultrafast speed is still a great challenge. Here we theoretically propose and experimentally demonstrate a reprogrammable plasmonic topological insulator, where the topological propagation route can be dynamically changed at nanosecond-level switching time, leading to an experimental demonstration of ultrafast multi-channel optical analog-digital converter. Due to the innovative use of electric switches to implement the programmability of plasmonic topological insulator, each unit cell can be encoded by dynamically controlling its digital plasmonic states while keeping its geometry and material parameters unchanged. Our reprogrammable topological plasmonic platform is fabricated by the printed circuit board technology, making it much more compatible with integrated photoelectric systems. Furthermore, due to its flexible programmability, many photonic topological functionalities can be integrated into this versatile topological platform.

## Abstract (web summary)

The development of fast and dynamic topological photonic platforms is an ongoing challenge. Here, the authors demonstrate a reprogrammable plasmonic topological insulator in which ultrafast electric switches allow for nanosecond-level switching time between different configurations.

## Introduction

The discovery of topological states of matter in condensed matter physics has inspired the search for analogous effects in classical and bosonic systems, especially fruitful in photonics. The beginnings of topological photonics can be traced to the efforts to emulate the quantum Hall effect (QHE), where the time-reversal symmetry of the system is broken by exploiting magneto-optic photonic crystals under external static magnetic fields^1–6^. However, magneto-optical effects in most magnetic materials are weak and in many cases, it can be cumbersome and inconvenient to implement a magnetic device. To overcome these limitations, nonmagnetic photonic topological systems are developed subsequently, in which the time-reversal symmetry is preserved, to mimic the quantum spin-Hall effect (QSHE) or quantum valley-Hall effect (QVHE)^7–11^. The unique topological features of light in topological photonics, such as unidirectional and backscattering-immune propagation against defects and sharp bends, offer a robust way to control the behavior of light. The study of topological photonics has revolutionized our understanding of light propagation, and holds great promise for practical photonic applications, especially for the development of robust linear and nonlinear photonic devices^12–16^.

Currently, the studies of photonic topological insulators (PTIs) are mainly focused on the development of different static photonic systems to realize a specific photonic topological phenomenon or functionality^17–19^, and hence their controllability and reconfigurability are limited. In practical applications, however, multiple photonic topological functionalities are expected to be achieved in a single but reconfigurable PTI, so that the time and costs associated with the design and fabrication process can be reduced. To this end, several key works on reconfigurable topological insulators^20–25^ have been reported recently, in which the reconfigurability is mostly implemented by changing either the geometry or material parameters. Unfortunately, these features in PTIs cannot be easily altered once the photonic structure has been fabricated. Moreover, the mechanical, thermal or optical method used to realize the reconfigurability is not convenient for use in compact photoelectric integrated systems, and the reconfiguring speed is limited. Recently, the emergence of programmable metasurfaces opens a digital way to dynamically control the metasurface functionality, which has spurred wide interest in metamaterial community^26–31^. However, limited by the reflection-type binary state, current programmable metasurfaces are mainly used to tailor the reflection and radiation of electromagnetic (EM) waves, and the study that aims to control the propagation route of guiding waves has been hardly reported.

In this work, taking advantage of both the flexible programmability of digital metasurfaces and the robust EM propagation of PTIs, we propose and experimentally demonstrate an ultrafast reprogrammable plasmonic topological insulator (RPTI), which is comprised of metallic elements arranged in a hexagonal lattice structure. To achieve an electrically driven programmability, each metallic element has six arms whose EM response can be electrically controlled via a positive-intrinsic-negative (PIN) diode, thus a topological bandgap can be created by breaking the space-inversion symmetry of the unit cell to emulate the QVHE. Moreover, each unit cell of the RPTI can be encoded independently to enable a dynamic control of topological light propagation routes.

Compared to existing controllable PTIs, our RPTI has two unique features. First, its programmability is achieved by electrically encoding the binary states of the PIN diode, leading to an ultrafast dynamic control of different topological propagation routes. Second, our RPTI is fabricated by a widely-used printed circuit board (PCB) technology, and thus it can be seamlessly integrated with the commonly used PCB-based photoelectric integrated circuits. These unique features are crucial for the development of versatile and intelligent topological photoelectric devices for future practical applications.

## Results

### The system

**Fig. 1:**
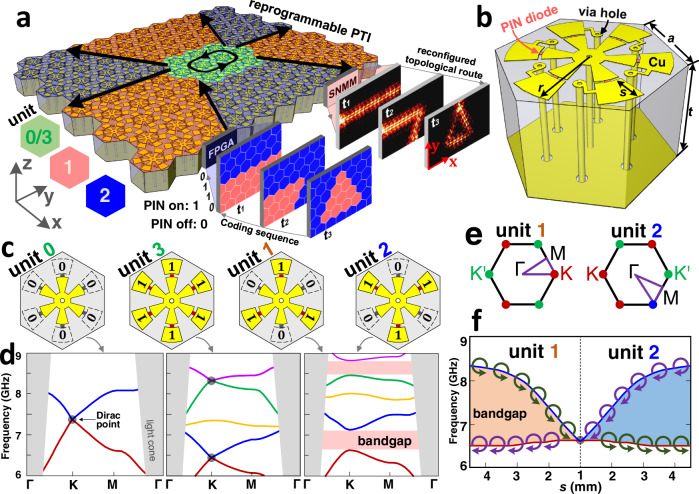
Design principle of RPTI. **a** Schematic of an ultrafast reprogrammable plasmonic topological insulator (RPTI). The digital state of each reprogrammable unit cell is controlled electrically by the field-programmable gate array (FPGA) controller, which enables one to dynamically reconfigure the topological propagation route as needed. **b** Reprogrammable unit cell. A six-arm metallic element and a ground plate perforated by six via holes are printed on the two facets of a dielectric spacer. The binary states of the metallic element are controlled by six positive-intrinsic-negative (PIN) diodes (shown in red). **c** Four digital states of a designed 2-bit reprogrammable unit cell, whose odd (even) outer arms hold the same binary state. **d** Band diagrams of a crystal with the designed 2-bit unit cell. **e** First Brillouin zone of digital unit-1 and unit-2. **f** Topological phase transition and the valley-chirality property of the first and second bands of digital unit-1 and unit-2. The valley bandgap is enlarged when increasing the length *s* of outer arm.

The proposed RPTI (Fig. 1a) consists of programmable unit cells (Fig. 1b) arranged in a hexagonal lattice, with each unit cell being in one of its four digital states “0”, “1”, “2”, and “3” (see Fig. 1c). This system configuration enables us to encode one RPTI into several distinct plasmonic crystal domains, as shown in Fig. 1a. Applying different coding sequences to each programmable unit cell at different times (*t*_1_, *t*_2_, and *t*_3_) using a field-programmable gate array (FPGA), we can dynamically reconfigure the size and pattern of each plasmonic crystal domain to realize an on-demand manipulation of the domain-wall interface along which the microwave propagates. Moreover, this reconfigurable topological propagation route can be observed directly in experiments using a scanning near-field microwave microscopy (SNMM). Specifically, the programmable unit cell in Fig. 1b consists of a dielectric spacer sandwiched between a controllable printed metallic element at the top and, at the bottom, a metallic ground plate perforated by six holes forming a hexagon. Unlike previous studies^32,33^, our disk has six inner arms and six outer arms, where each inner arm is bridged with one outer arm via a PIN diode. Furthermore, to ensure that the direct current (DC) bias voltage of the inner arms is zero, the six inner arms are connected to the ground plate by a metallic via hole. The six outer arms are connected to a FPGA control network by six metallic via holes. As a result, applying different DC voltages to each outer arm, one can dynamically switch “on” and “off” the corresponding PIN diode to emulate the binary states “1” and “0”, respectively. The geometry parameters of our RPTI studied in this work are $a=15/\sqrt{3}$ mm, *t* = 3 mm and *r* = 7.5 mm (see Fig. 1b). In addition, the radius of the inner metallic circular disk is 1.5 mm, and the gap distance between the inner and outer arms is 0.3 mm.

In principle, the reprogrammable unit cell in Fig. 1b is a 6-bit digital element possessing 2^6^ = 64 digital states. To simply our discussion, the odd (even) outer arms are designed to be in the same binary state as detailed in Supplementary Fig. 1, so that the 6-bit digital element can be reduced to a 2-bit digital element possessing 2^2^ = 4 digital states. In the context of programmable metasurfaces^31^, they could be naturally encoded as 0 = “00”, 1 = “01”, 2 = “10” and 3 = “11”, as listed in Fig. 1c. Here, the binary codes “1” and “0” mean that the PIN diode is switched “on” and “off”, respectively. In the following, we denote each digital state as a digital unit in Fig. 1c, though in practice they are the same programmable unit cell. Our RPTI is designed in the microwave regime, where metallic loss can be suppressed and spoof surface plasmons (SSP) exist^34^. As demonstrated in Supplementary Figs. 2-5, the field of SSP is concentrated at the outer arm, if the corresponding PIN diode is switched on. However, if the diode is switched off, the field of SSP is located at the inner arm, and the corresponding outer arm is “invisible” to EM waves^35^. Based on this feature, we can engineer and dynamical control the band diagram of the RPTI, as demonstrated in Fig. 1d. In particular, due to the *C*_*6*_ symmetry of the unit cell, there is a Dirac point in the band diagram of digital unit-0, in which all diodes are switched off. Similarly, Dirac points also appear in the band diagram of digital unit-3, in which all diodes are switch on. For the digital unit-1, the diodes on its odd (even) arms are switched off (on), so the *C*_*6*_ symmetry is reduced to *C*_*3*_ symmetry. As a result, the *C*_*6*_-symmetry-protected Dirac points are gapped out to form bandgaps at **K** and **K′** valley points, as illustrated in the band diagram of digital unit-1.

The emergence of valley-Hall states within the bandgap, which underpins the connection between the observed phenomena and applications on one side and the topological invariants characterizing the system on the other, can be explained as follows: as digital unit-1 and unit-2 are related by a π/3 in-plane rotation in real space, the first Brillouin zone (FBZ) of digital unit-2 in momentum space can also be obtained from that of digital unit-1 via a π/3 rotation (Fig. 1e). As a result, the **K**(**K′**) valleys of unit-1 are transformed to **K**′(**K**) valleys of unit-2. Since the valley Chern numbers of **K** and **K′** are ±1/2^33,36,37^, for a domain-wall interface between a unit-1-type domain and a unit-2-type domain, the difference of the valley Chern number across the interface for **K** and **K′** is ±1. According to the principle of bulk-edge correspondence^36,37^, a valley Hall edge mode band would emerge in the valley bandgap. In Fig. 1f, we further show that this nontrivial valley bandgap can be broadened by increasing the length *s* of the outer arm. By switching the “on” and “off” states of the PIN diode, the binary expression of topological phase transition between unit-1 and unit-2 domains is encoded as the digital expression, which leads to a digital and dynamically controllable topological propagation interface. In condensed matter physics, manipulating the valley Hall states in a dynamical way is challenging and as such we believe that our work could inspire renewed research efforts in programmable valleytronics^38^ in condensed matter physics.

### Projected bands of domain-wall interfaces

**Fig. 2:**
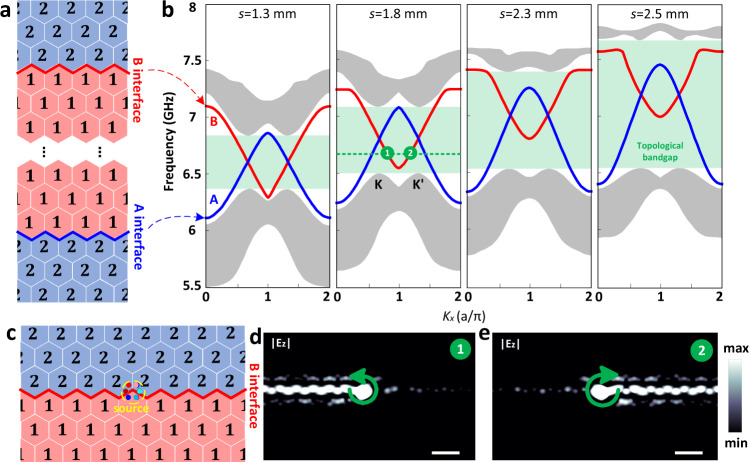
Domain-wall interface modes of RPTI and the unidirectional topological propagation feature. **a** Topological domain-wall interface. The interfaces A and B separate two crystal domains, which are filled with digital unit-1 (symbol “1”) and unit-2 (symbol “2”), respectively. **b** Projected band diagrams. The topological bandgap increases with the length s of outer arm. **c** Straight waveguide with a B-type nontrivial interface, where a chiral source is employed. **d**, **e** Simulated unidirectional propagations with the RCP (**d**) and LCP (**e**) excitations. White scale bar, 30 mm.

To demonstrate the topological nature of nontrivial domain-wall interface modes, we encode the unit cells of RPTI so as to construct two valley-Hall domain-wall interfaces with zigzag shape, namely the interfaces A and B in Fig. 2a, which separate two domains filled with digital unit-1 and unit-2, respectively. As demonstrated in Fig. 1f, the size of the valley bandgap is tunable with respect to the length s of the outer arm. Here the projected band diagrams corresponding to different lengths *s* are given in Fig. 2b. In each case, there are two valley-Hall interface modes. The blue (red) solid line indicates the nontrivial interface mode of the interface A (B), and the gray-shaded regions represent the projected bulk modes. From physical point of view, the valley bandgap should be as small as possible so as to differentiate it from a glide-plane symmetric interface^39^. However, in order to achieve a device with improved functionality, one should aim to achieve a large bulk topological bandgap that can well preserve the topological properties of the valley-Hall states. After a careful consideration of the tradeoffs between achieving a good valley-Hall phase and a broad topological spectral range, we found out that the optimum value of the length *s* is 1.8 mm.

To study the property of unidirectional propagation of topological interface modes, a straight RPTI waveguide with the B-type interface is encoded in Fig. 2c, where a chiral source consisting of six electric dipoles with ±π/3 phase difference between adjacent dipoles is employed to excite a right-circularly polarized (RCP) or left-circularly polarized (LCP) wave. As shown in Fig. 2b, if the source has frequency of 6.7 GHz, two distinct waveguide modes with opposite group-velocity, corresponding to points 1 and 2, are generated. Furthermore, from the valley-chirality-locking property of the topological interface modes, the chirality of the interface mode at point 1 is also opposite to that at point 2. Consequently, a unidirectional propagation of microwave can be achieved by selectively exciting an RCP or LCP phase vortex at the source point in Fig. 2c, which is numerically demonstrated in Fig. 2d, e.

### Reconfigurable topological light propagation

**Fig. 3:**
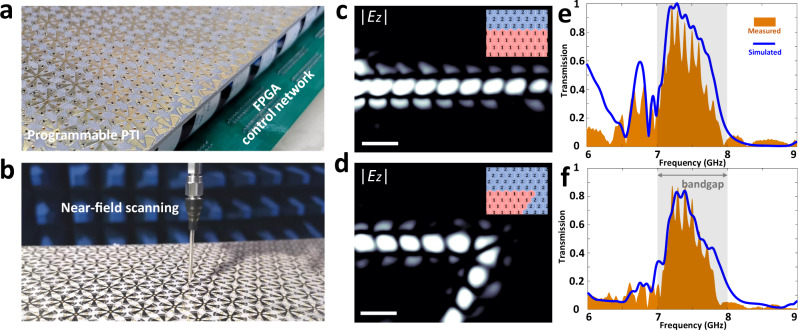
Experimental results of reprogrammable topological propagation routes. **a** Photograph of the fabricated RPTI. **b** Experimental setup of near-field scanning. **c**, **d** Measured near-field distributions of |*E*_*z* _| in topological straight and 120-degree-bend waveguides at 7.2 GHz, respectively. The corresponding coding patterns are depicted in insets. White scale bar, 30 mm. **e**, Comparison of measured and simulated transmissions of a topological straight waveguide. **f** Comparison of measured and simulated transmissions of a topological 120-degree-bend waveguide.

In addition to the unidirectional propagation, the feature of immunity against sharp bends is also experimentally studied here. As illustrated in Fig. 3a, a fabricated RPTI is dynamically controlled using an FPGA control network. To verify the backscattering-immune propagation against sharp bends, different topological and non-topological propagation routes are programmed in our RPTI by properly encoding each of its unit cells via the FPGA controller, and the corresponding near-field distributions of |*E*_*z* _| are measured by a SNMM experimental setup (see Fig. 3b).

To be more specific, a straight-waveguide propagation route is studied first with the measured near-field distribution of |*E*_*z* _| at 7.2 GHz (~0.5 GHz frequency shift with respect to simulated results) presented in Fig. 3c. The corresponding coding pattern used to implement the straight propagation route is indicated in inset. Moreover, the simulated and measured transmissions of the straight topological propagation route are compared in Fig. 3e, where a good agreement is observed. In addition to the straight propagation route, we further study the case of a 120-degree-bend propagation route. As shown in Fig. 3d, the propagation remains immune to the sharp bend, and the corresponding transmission spectra are given in Fig. 3f. It shows that the transmission of a 120-degree-bend topological interface only decreases slightly with respect to that of a straight topological interface. In addition to the robustness against the sharp bending of propagation routes, it is also relevant to study the effect of defects and disorder, which could generally arise from manufacturing imperfections and operational failure of devices. Our study shows that the proposed topological interface is rather robust even in the presence of high levels of disorder (for details see Supplementary Fig. 7).

### Ultrafast multi-channel optical analog-digital converter

**Fig. 4:**
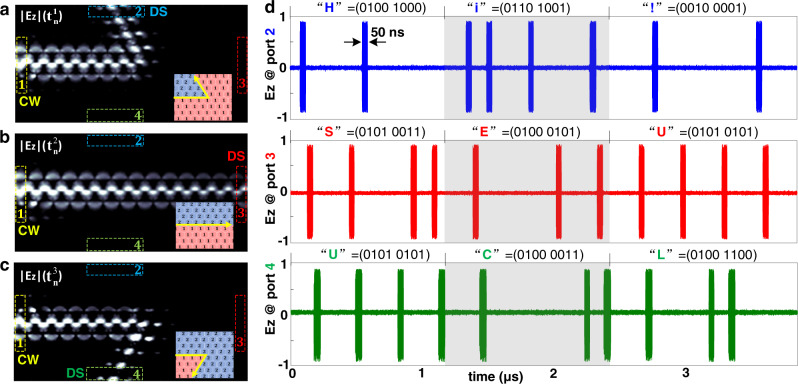
Proof-of-principle experiment of a reprogrammable multi-channel optical analog-digital converter. **a**–**c** Encoded propagation routes and the corresponding simulated field distributions of |E_z _| as a function of time tn. Here, *t*_*n*_^1^ = *t*_0_ + 3*n*∆*t*, *t*_*n*_^2^ = *t*_0_ + (3*n* + 1)∆*t*, and *t*_*n*_^3^ = *t*_0_ + (3*n* + 2)∆*t*, where *n* = 0,1,2,3,…, ∆*t* is the switching period, and *t*_0_ is the start time of the coding. A continuous wave (CW) is pumped at the input port 1. Because of the high-speed switching of topological propagation routes, the input CW is modulated directly as a digital signal (DS) detected at the output port. **d** Measured output signal of each port. Three ASCII sequences (i.e. “Hi!”, “SEU”,“UCL”) are modulated and transmitted in our RPTI, and the switching time of topological propagation route can be as short as 50 ns.

Due to the flexible programmability, many existing photonic topological functionalities can be readily implemented in our universal RPTI platform. More importantly, our RPTI can also be used to achieve some dynamic functionalities that cannot be implemented in static PTIs or low-speed reconfigurable PTIs. Here, a proof-of-principle experiment of a topologically protected multi-channel optical analog-digital converter with ultrafast control is demonstrated in Fig. 4. To simplify our demonstration, four waveguide ports are designed in our RPTI as illustrated in Fig. 4a–c, and a continuous wave (CW) with frequency 7.2 GHz is pumped at the input port 1. By encoding different topological propagation routes, we can dynamically switch the output port from ports 2 to port 3 or port 4, thus the input CW analog signal can be directly discretized into different digital signals (DS) at the output ports. To demonstrate this remarkable feature, the input CW signal is modulated as three ASCII sequences (the corresponding glyph sequences are “Hi!”, “SEU”, and “UCL”), and the three output digital signals are measured experimentally, as shown in Fig. 4d. The measured results show that the switching time in this system is as short as 50 ns, namely more than seven orders of magnitude faster than that of previous work^20^. Moreover, since only one topological propagation channel exists at each time step, the interference among the output ports is negligible, even when the output ports are closed to each other. This low cross-talk feature is crucial in practical high-fidelity digital communications.

In principle, the reprogrammable plasmonic topological insulator demonstrated here can be viewed as a programmable photonic circuit. In the current state-of-the-art photonic technology, the dynamic programmable functionality of most photonic integrated circuits (PICs) is mainly achieved by locally switching analog optical gates^40^, which in turn are usually controlled mechanically, thermally or electrically. As a result, the number and physical location of output ports are fixed once the PIC is fabricated. Since the dynamic switching functionality of our PTI is implemented by tuning its global topology, the tunable degree of freedom of the proposed PIC is much higher than that of current programmable photonic circuits. This translates in a more flexible and agile design of light propagation routes. However, facilitated by advanced fabrication methods (such as MEMS, CMOS etc.), the current programmable PIC is higher in integration and lower in energy consumption than our PTI. Thus, in the future, it will be a promising and important work to further reduce the energy consumption or improve the integration of the proposed PTI by using advanced fabrication methods.

## Discussion

To summarize, we have theoretically proposed and experimentally demonstrated a reprogrammable plasmonic topological insulator with ultrafast switching speeds at nanosecond scale. By investigating the band diagrams of the proposed RPTI, we shown that a topological bandgap emulating QVHE can be created by digitally encoding its unit cells via switching electrically the PIN diodes “on” and “off”. As each unit cell can be independently programmed, it was experimentally demonstrated that valley-Hall topological light propagation routes in our RPTI can be dynamically reconfigured. Moreover, we experimentally implemented a topologically protected multi-channel optical analog-digital converter to illustrate the ultrafast control feature of our RPTI, and demonstrated that the switching time in our case is as short as 50 ns, which is more than seven orders of magnitude faster than that of previous work. Our work brings the current studies of photonic topological insulators to a digital and intelligent era, which could open an avenues towards the development of software-defined photoelectric elements in high-speed communications and computation-based intelligent devices with built-in topological protection.

## Methods

### Numerical simulations

The numerical simulations were performed using the finite-element method based software COMSOL Multiphyiscs. In Fig. 1b, the metallic material (yellow) is copper and the dielectric substrate (semitransparent gray) is a widely-used commercial PCB board (F4B), whose relative permittivity is 2.65 and loss tangent is 0.001. The PIN diodes (bold pink) are modeled by lumped elements^31,41^. The “off” state is modeled by an inductor-capacitor series circuit, where the inductance *L* = 0.4 nH and the capacitance *C* = 40 fF. The band diagrams in Fig. 1d were evaluated at the outer-arm length *s* = 1.7 mm. The “on” state is modeled by a resistor-inductor series circuit, where the resistance *R* = 2.2 Ω and the inductance *L* = 0.32 nH. The projected band diagrams in Fig. 2a, b were evaluated using a supercell containing 10 unit cells on either side of the domain wall, and periodic boundary conditions were imposed at left and right sides.

### Sample fabrication

Our RPTI sample was fabricated in multiple-layer lamination process of PCB technique. In the layer stack management, as presented in Supplementary Fig. 1b, L1 and L2 are printed on the top and bottom facets of a F4B substrate (3 mm thickness), whereas *L*_3_ and bias-voltage-line layers (Supplementary Fig. 6b) are printed on the top and bottom facets of a FR4 substrate (0.5 mm thickness), whose relative permittivity is 4.3 and loss tangent is 0.025. A prepreg layer (FR4 with 0.2 mm thickness) for bonding the F4B and FR4 substrates is used between *L*_2_ and *L*_3_ layers. The thickness of the printed copper film in each layer is 35 µm. The overall size of our RPTI is ~465 × 221 mm^2^, containing 488 digital units with 2928 PIN diodes. Commercially available Aluminum Gallium Arsenide PIN diodes (MADP-000907-14020) are employed in the fabrication.

### Experimental measurements

A scanning near-field microwave microscopy is employed to measure the near electric fields and transmissions. Our SNMM is composed of vector network analyser (Agilent N5230C), low-noise amplifier, and several phase-stable coaxial cables. Two coaxial probes that act as the emitter and receiver are employed in measurements. One probe is soldered on the edge of the RPTI to generate a plasmonic mode, whereas the other one is fixed on a scanning support to scan the near-field distributions. During the measurements, the RPTI is placed on an acrylic supporter, as demonstrated in Supplementary Fig. 6a. Since each digital unit requires two voltage-control signals to realize the 2-bit states (Supplementary Fig. 1), our RPTI has 976 independent channels for voltage control. In order to dynamically steer the coding pattern of our RPTI, we use three field programmable gate arrays with a total of 1200 digital-voltage channels. The PIN diodes are connected to FPGA through white flexible printed circuit (FPC) cables, as shown in Supplementary Fig. 6c. In the measurement of the multi-channel optical analog-digital converter, a high-frequency oscilloscope (Agilent DSO9013A) and a signal generator (Agilent E8267D) are used to measure the wave forms in the time domain.

## Supplementary information

Supplementary Information

## Data Availability

The data in Figs. 1–4 that support the findings of this study are available from the corresponding author upon reasonable request.

## Code availability

The code that supports the findings of this study are available from the corresponding author upon reasonable request.
