# Supplementary material for: Reprogrammable plasmonic topological insulators with ultrafast control
Source: Nat Commun. 2021 Sep 15;12:5468. doi: 10.1038/s41467-021-25835-6 (PMC8443663; doi:10.1038/s41467-021-25835-6)
Supplement: Supplementary file 1 — Supplementary Information [file 41467_2021_25835_MOESM1_ESM.pdf]

Supplementary Information: Reprogrammable plasmonic topological insulators with ultrafast control

Jian Wei You[1,2,3], Qian Ma[2,3], Zhihao Lan[1], Q. Xiao[2], Nicolae C. Panoiu[1*], and Tie Jun Cui[2*]

[1]Department of Electronic and Electrical Engineering, University College London,
Torrington Place, London WC1E 7JE, United Kingdom.
[2]State Key Laboratory of Millimeter Waves and Institute of Electromagnetic Space, Southeast University,
Nanjing 210096, China.
[3]These authors contributed equally: Jian Wei You, Qian Ma
[*]Email: tjcui@seu.edu.cn, n.panoiu@ucl.ac.uk

ABSTRACT

This Supplementary Information section includes Supplementary Figures 1-8 and their descriptions.

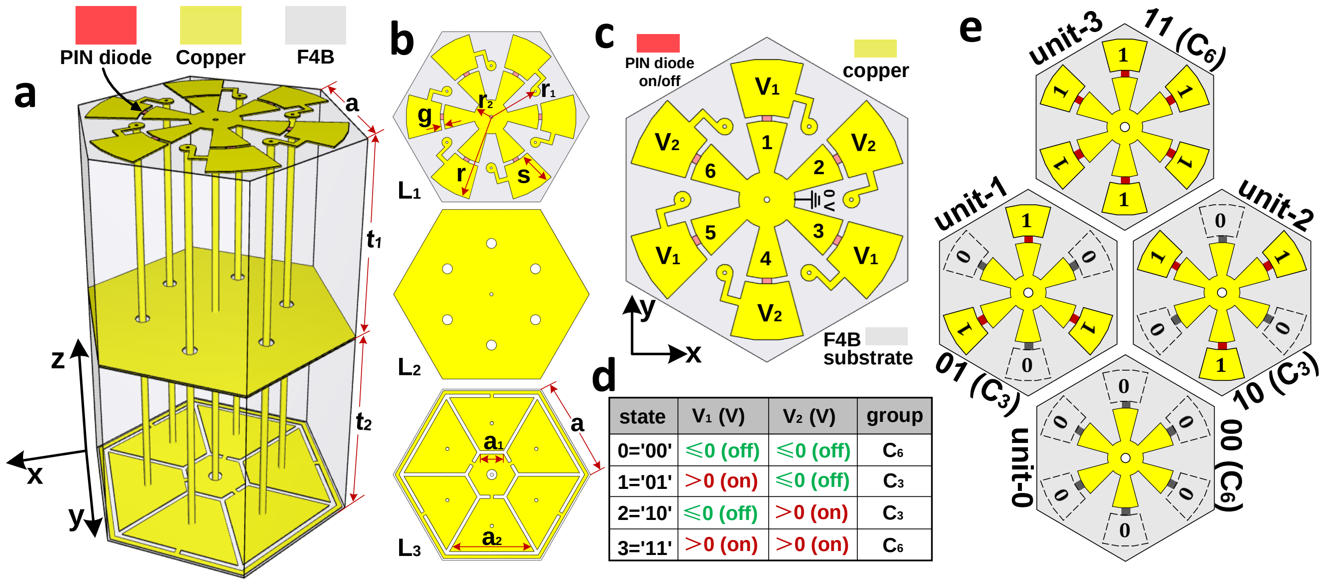

state	V1 (V)	V2 (V)	group
0='00'	⩽0 (off)	⩽0 (off)	C6
1='01'	>0 (on)	⩽0 (off)	C3
2='10'	⩽0 (off)	>0 (on)	C3
3='11'	>0 (on)	>0 (on)	C6

Supplementary Fig. 1. | Design of the 2-bit unit cell. a, Programmable 2-bit unit cell. Three metallic layers separated by two layers of dielectric material (F4B) are connected by seven metallic via holes. The edge length of the hexagonal unit cell is $a = 15/\sqrt{3}$ mm. The thickness of dielectric layers are $t_1 = 3$ mm and $t_2 = 0.2$ mm, respectively. **b**, Designed six-arm metallic disk with six PIN diodes bridging its inner and outer arms printed on the top layer (L_1). The structure parameters are $r = 7.5$ mm, $r_1 = 4.5$ mm, $r_2 = 1.5$ mm, $s = 2.5$ mm, and $g = 0.3$ mm. A metallic layer (L_2) is designed as a zero-voltage ground plate, where the radius of the big and small holes are 0.8 mm and 0.6 mm, respectively. In order to ensure that the voltage of odd (even) outer arms is the same, the bottom layer (L_3) is designed to connect the outer arms. The structure parameters of the pattern on L_3 are $a_1 = 2.08$ mm, and $a_2 = 7.45$ mm. **c**, **d**, Encoding of 2-bit unit cell. The voltage of inner arms is set to zero, whereas the odd (even) outer arms possess the same voltage and their voltage is V_1 (V_2). The PIN diode is switched on (binary state "1") when V_1 (or V_2) > 0, and the PIN diode is switched off (binary state "0") when V_1 (or V_2) ≤ 0. The four states of the 2-bit unit cell are renamed as digital units 0, 1, 2, and 3. **e**, Schematic illustration of the four digital states of a designed 2-bit unit. When the PIN diode is switched on, the corresponding inner and outer arms are connected. Whereas, the bridge of the electric current between the inner and outer arms is cut off, when the PIN diode is switched off.

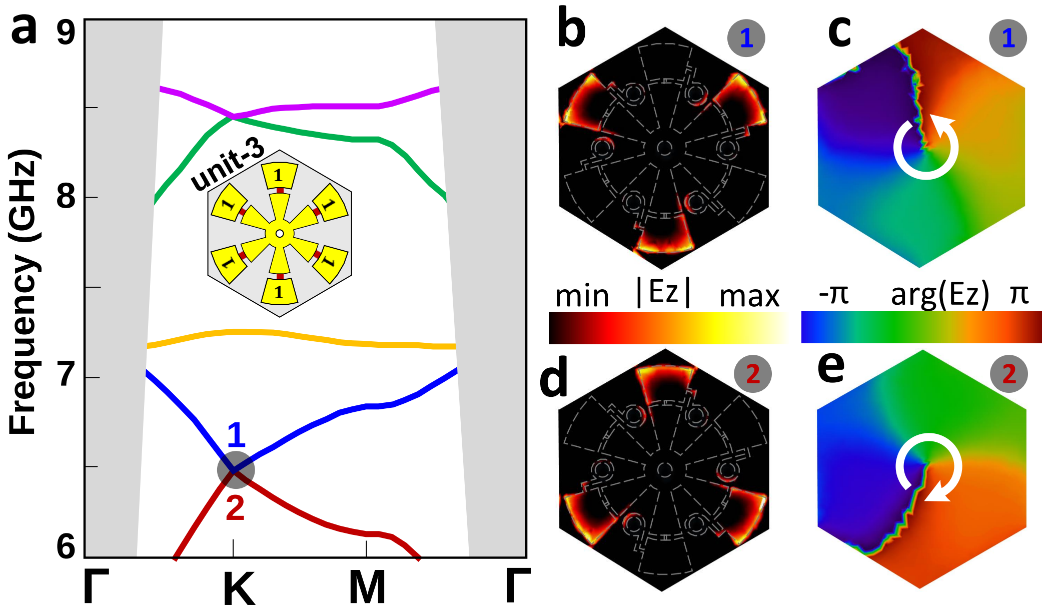

Supplementary Fig. 2. | Optical responses of the digital unit-3. a, Photonic band structure. As illustrated by the inset, all diodes are switched on, thus the digital unit-3 belongs to the C_6 point symmetry group. Due to the C_6 symmetry-inversion protection, two Dirac cones exist at the K point in the band diagram. **b**-**e**, The electric field distributions of $|E_z|$ (**b**,**d**) and the corresponding phase distributions (**c**,**e**) of the degenerate eigenmodes at the points "1" and "2" in **a**. Due to the plasmon-induced field confinement effect, the electric field is confined around the outer arms as all diodes are switched on. As shown in **c** and **e**, the eigenmodes at the points "1" and "2" have opposite chirality.

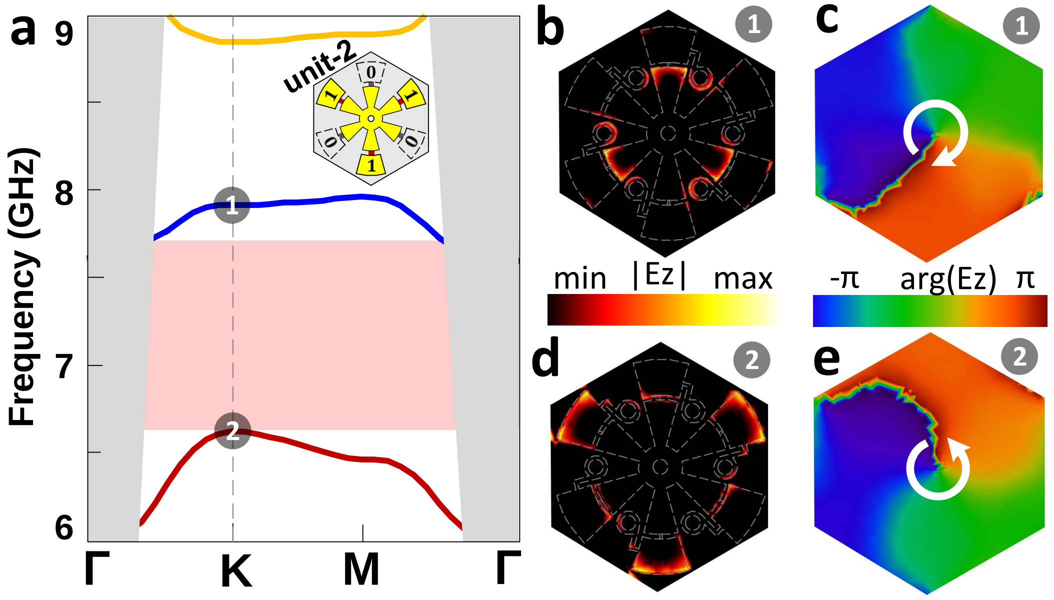

Supplementary Fig. 3. | Optical responses of the digital unit-2. a, Photonic band structure. As illustrated by the inset, the diodes on the odd (even) arms are switched off (on), thus the digital unit-2 belongs to the C_3 point symmetry group. Due to the breaking of the C_6 symmetry-inversion protection, the Dirac point is gapped out and a bandgap emerges at the K valley point. **b**-**e**, The electric field distributions of $|E_z|$ (**b**,**d**) and the corresponding phase distributions (**c**,**e**) of eigenmodes at the points "1" and "2" in **a**. The field distribution in **b** shows that the electric field is confined to the odd inner arms and the corresponding metallic outer arms are "invisible" for microwave, because the bridge of microwave current is terminated when the corresponding diode is switched off. On the contrary, the electric field in **d** is confined to the even outer arms as the corresponding diodes are switched on. Moreover, the eigenmodes belonging to the first and second band have opposite chirality.

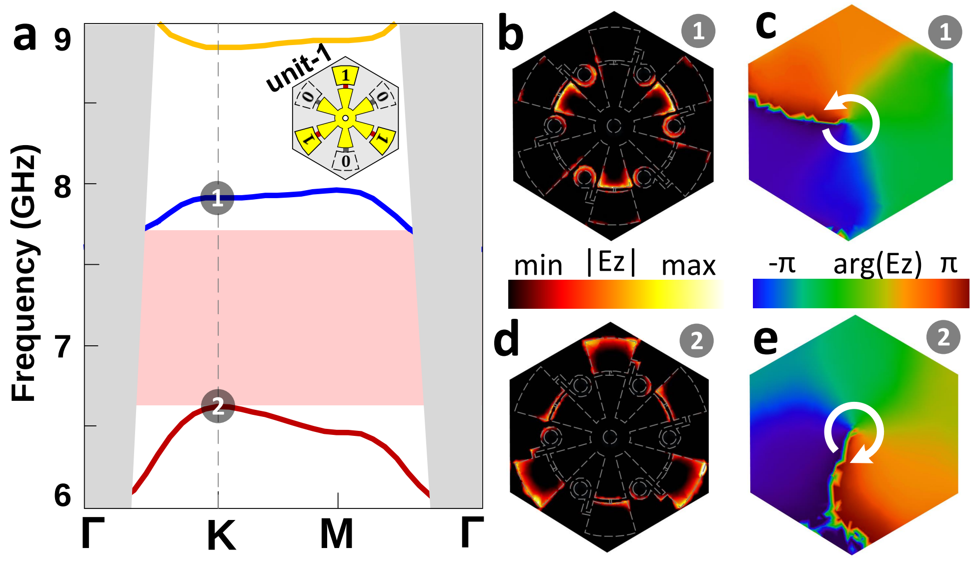

Supplementary Fig. 4. | Optical responses of the digital unit-1. a, Photonic band structure. As illustrated by the inset, the diodes on the even (odd) arms are switched off (on), thus the digital unit-1 belongs to the C_3 point symmetry group. Due to the breaking of the C_6 symmetry-inversion protection, the Dirac point is gapped out and a bandgap emerges at the K valley point. **b**-**e**, The electric field distributions of $|E_z|$ (**b**,**d**) and the corresponding phase distributions (**c**,**e**) of eigenmodes at the points "1" and "2" in **a**. The field distribution in **b** shows that the electric field is confined to the even inner arms and the corresponding metallic outer arms are "invisible" for microwave, because the bridge of microwave current is terminated when the corresponding diode is switched off. On the contrary, the electric field in **d** is confined to the odd outer arms as the corresponding diodes are switched on. The eigenmodes belonging to the first and second band have opposite chirality as shown in **c** and **e**. More importantly, the valley chirality of the first and second bands of the digital unit-1 is reversed as compared to that of the digital unit-2 (see Supplemenatry Figs. 3c and 3e). Thus, there is a topological phase transition between the digital units 1 and 2.

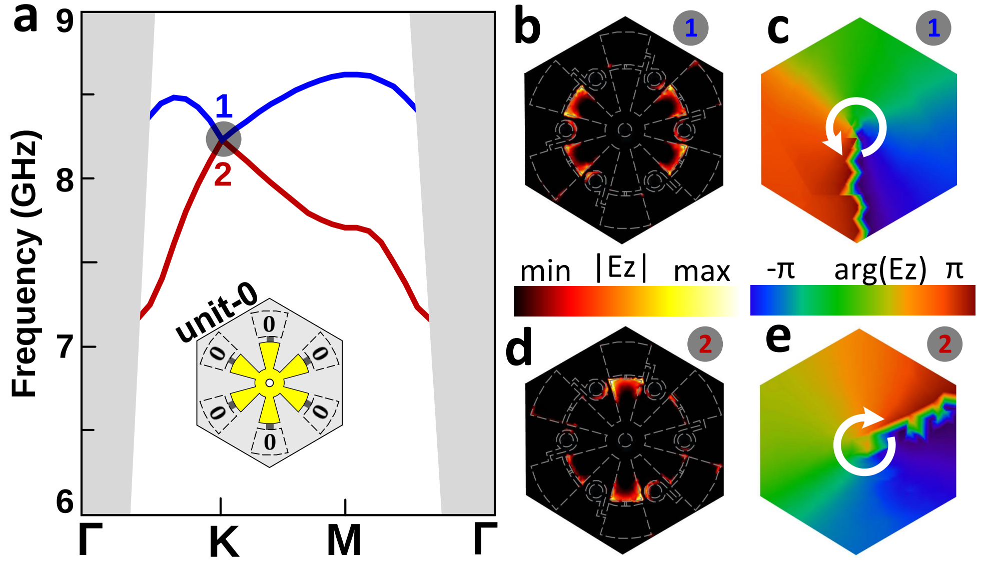

Supplementary Fig. 5. | Optical responses of the digital unit-0. a, Photonic band structure. As illustrated by the inset, all diodes are switched off, thus the digital unit-0 belongs to the C_6 point symmetry group. Due to the C_6 symmetry-inversion protection, a Dirac cone exists at the K point. **b**-**e**, The electric field distributions of $|E_z|$ (**b**,**d**) and the corresponding phase distributions (**c**,**e**) of degenerate eigenmodes at points "1" and "2" in **a**. Due to the plasmon-induced field confinement effect, the electric field is confined around the inner arms as all diodes are switched off. As shown in **c** and **e**, the eigenmodes at the points "1" and "2" have opposite chirality.

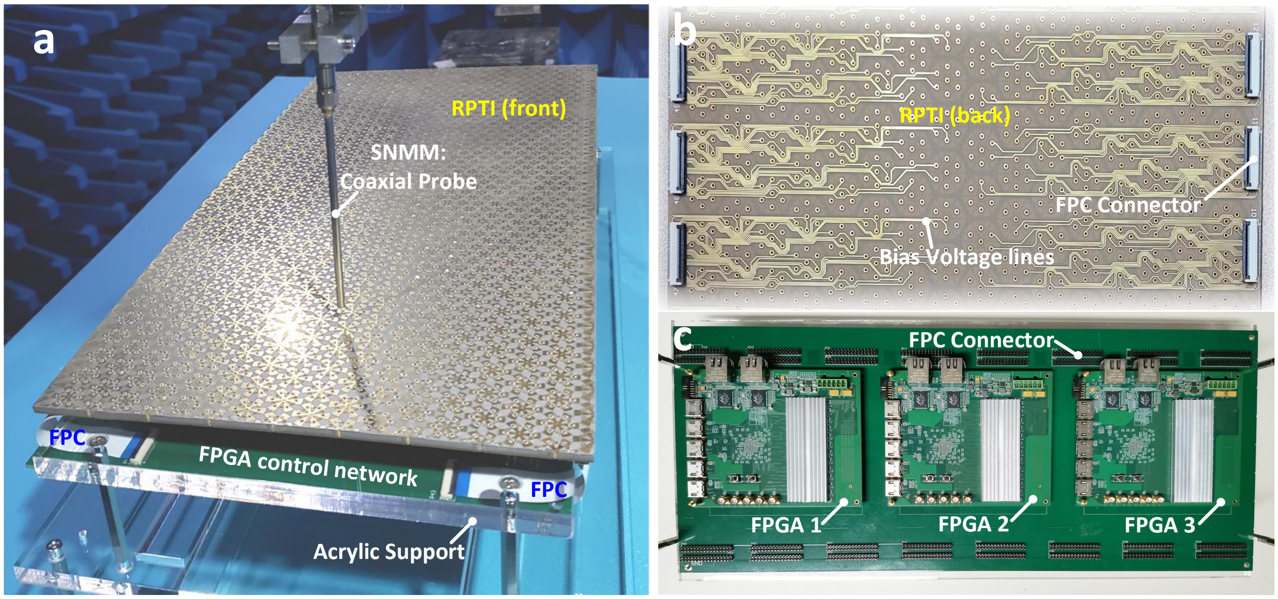

Supplementary Fig. 6. | Experimental measurements of near-field distribution. a, Near-field measurement system based on a scanning near-field microwave microscopy (SNMM). A coaxial probe is fixed on a scanning support, and it is vertical to the measured RPTI. The distance between the detector probe and the RPTI surface is 2 mm. FPGA and bias-voltage circuit are installed below the RPTI, and fixed in an acrylic support. **b**, The back of the RPTI, on which the bias voltage lines are printed to control PIN diodes. In experiments, 16 digital units are grouped as a single set, which is connected to an FPC connector with 32 channels. **c**, The FPGA control network composed of three FPGA modules. An FPGA module independently controls 400 voltage channels.

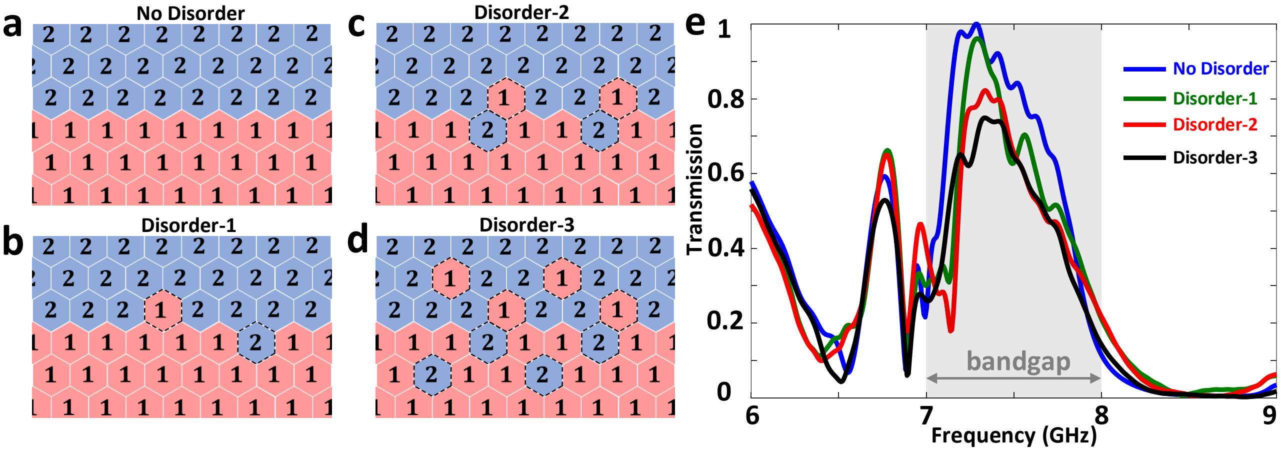

Supplementary Fig. 7. | Robustness against disorder. a, Straight light propagation route in the topological case without disorder. **b-d**, Straight light propagation routes in the topological case with different disorder strengths. **e**, Numerically computed transmission spectra pertaining to straight light propagation routes in the topological case with different disorder strengths. Although the transmission decreases as the degree of disorder increases, the proposed topological interface is still robust even in the presence of high levels of disorder.

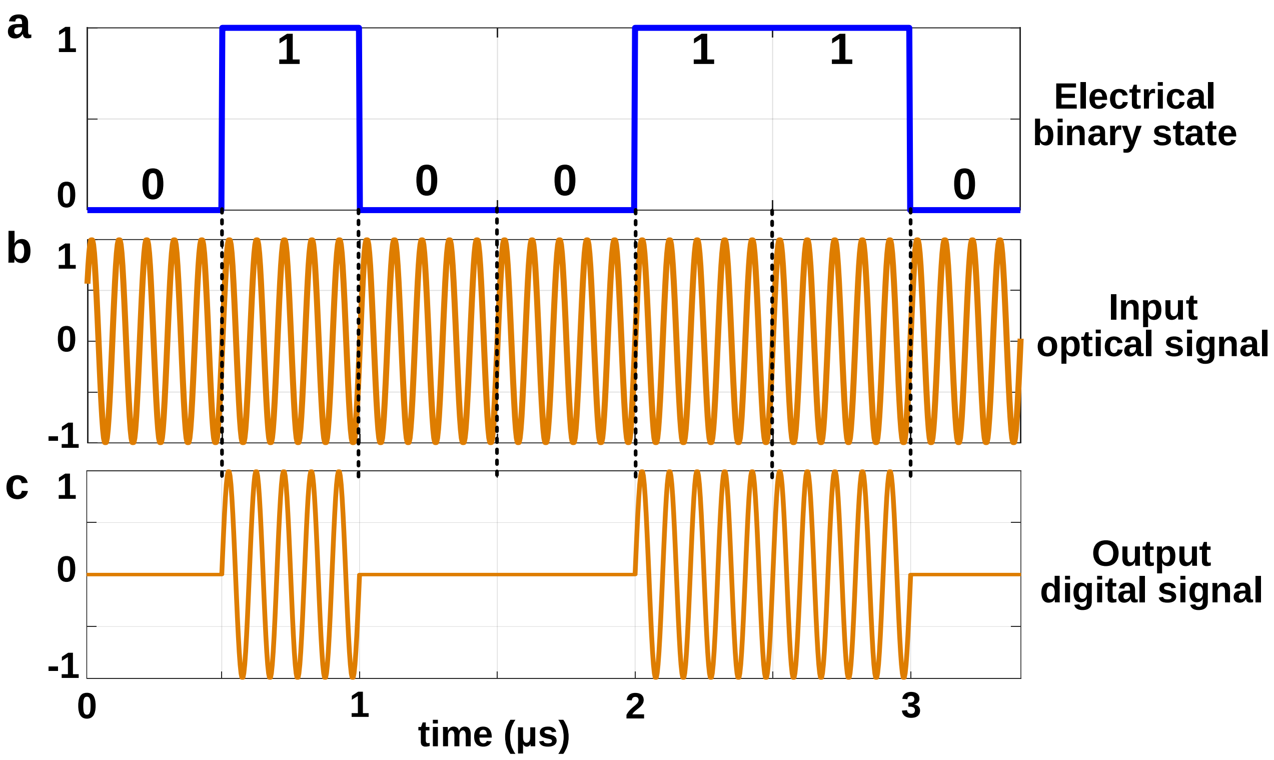

Supplementary Fig. 8. | Schematic diagram of optical analog-digital converter. a, Electrical binary code sequence as a function of time, in which "0" and "1" states indicate that the corresponding output ports are opened and closed, respectively. **b**, Optical continuous wave is pumped at the input port. **c**, Output digital signal. Since the electrical binary code sequence controls whether the output port is opened or closed, the input optical analog signal is converted at the output to a digital signal. This optical modulation is known as the amplitude shift keying (ASK).
